# Genetic and Functional Analysis of the *pks* Gene in Clinical Klebsiella pneumoniae Isolates

**DOI:** 10.1128/spectrum.00174-23

**Published:** 2023-06-21

**Authors:** Chenshuo Luo, Yanshu Chen, Xueni Hu, Shanjian Chen, Yulan Lin, Xiaoqian Liu, Bin Yang

**Affiliations:** a Department of Laboratory Medicine, the First Affiliated Hospital, Fujian Medical University, Fuzhou, Fujian Province, China; b Department of Laboratory Medicine, National Regional Medical Center, Binhai Campus of the First Affiliated Hospital, Fujian Medical University, Fuzhou, Fujian Province, China; c Fujian Key Laboratory of Laboratory Medicine, The First Affiliated Hospital, Fujian Medical University, Fuzhou, Fujian Province, China; d Gene Diagnosis Research Center, The First Affiliated Hospital, Fujian Medical University, Fuzhou, Fujian Province, China; University of Maryland Eastern Shore

**Keywords:** *pks* island, genotoxins, colibactin, *Klebsiella pneumonia*, virulence

## Abstract

The *pks* gene cluster encodes colibactin, which can cause DNA damage and enhance the virulence in Escherichia coli. However, the role of the *pks* gene in Klebsiella pneumoniae has not been fully discussed. The aim of this study was to analyze the relationship between the *pks* gene cluster and virulence factors, as well as to assess antibiotic resistance and biofilm formation capacity in clinical isolates of Klebsiella pneumoniae. Thirty-eight of 95 clinical K. pneumoniae strains were *pks* positive. *pks*-positive strains usually infected emergency department patients, and *pks*-negative strains often infected hospitalized patients. The positive rates of K1 capsular serotype and hypervirulence genes (*peg-344*, *rmpA*, *rmpA2*, *iucA*, and *iroB*) were significantly higher in the *pks*-positive isolates than the *pks*-negative isolates (*P* < 0.05). The biofilm formation ability of *pks*-positive isolates was stronger than that of *pks*-negative isolates. Antibacterial drug susceptibility test showed the resistance of *pks*-positive isolates was weaker than that of *pks*-negative isolates. In conclusion, patients with *pks*-positive K. pneumoniae infection might have worse treatment outcomes and prognosis. *pks*-positive K. pneumoniae might have stronger virulence and pathogenicity. Clinical infection with *pks*-positive K. pneumoniae needs further attention.

**IMPORTANCE** The infection rate with *pks*-positive K. pneumoniae has been increasing in recent years. Two previous surveys in Taiwan reported 25.6% *pks* gene islands and 16.7% *pks*-positive K. pneumoniae strains in bloodstream infections, and Chinese scholars also did a survey of K. pneumoniae bloodstream infections in Changsha, China, and found 26.8% *pks*-positive K. pneumoniae. In addition, it was found that the *pks* gene cluster might encode colibactin, which could be related to the virulence of K. pneumoniae. Studies confirmed that the prevalence of colibactin-producing K. pneumoniae was increasing. It is necessary to consider the clear relationship between the *pks* gene cluster and high pathogenicity in K. pneumoniae.

## INTRODUCTION

Klebsiella pneumoniae is an opportunistic pathogen with an increasing incidence in infectious diseases. Currently, highly virulent and highly resistant K. pneumoniae strains are prevalent worldwide. Compared to classic K. pneumoniae (cKP), hypervirulent K. pneumoniae (hvKP) is more likely to cause community-acquired infection, leading to liver abscess, pneumonia, and other diseases (1). In recent years, the proportion of carbapenem-resistant hypervirulent K. pneumoniae (CR-hvKP) has also increased, which could result in the failure of clinical anti-infective treatment or prolongation of the infectious disease course (2, 3). These changes pose significant challenges to clinical treatment. Therefore, the characteristics of their pathogenicity and the expression of virulence genes are increasingly attracting attention.

It has been shown that the five virulence genes *iucA* (aerobactin siderophore), *rmpA* (hypermucositity), *rmpA2* (yersiniabactin), *iroB* (salmochelin siderophore), and *peg-344* (putative transporter) can be used to distinguish between hvKP and cKP with a high degree of accuracy (≥95%) (4). Certain serotypes, such as K1, K2, K5, K20, K54, and K57, are strongly linked with invasive infections in the host. The K1 and K2 serotypes of K. pneumoniae, in particular, can develop drug-resistant phenotypes by mediating various drug-resistant genes via mobile genetic elements, posing a major challenge for clinical treatment (5, 6).

The polyketide synthase (*pks*) gene cluster, which encodes the synthetic genotoxin colibactin, has been primarily found in *Enterobacteriaceae*, such as Escherichia coli, K. pneumoniae, and *Citrobacter* (7, 8). The *pks* gene cluster encodes a synthetic genotoxin of colibactin that induces DNA damage in eukaryotic cells, which is associated with other bacterial virulence factors (adhesins, toxins, and siderophores) (7, 9). Studies have found that *pks*-positive K. pneumoniae infection exacerbated lymphopenia in septic mouse models (10), promotes the development of meningitis (11), and significantly increases mortality in patients (12). Therefore, there might be a potential correlation between *pks* gene clusters and virulence.

In this study, we collected a total of 98 clinical isolates of K. pneumoniae from our hospital and analyzed the prevalence of the *pks* gene cluster and virulence genes, antimicrobial susceptibility, and biofilm formation. This study aimed to evaluate the effect of *pks* gene cluster on pathogenicity and virulence in order to provide new insights for the clinical treatment of K. pneumoniae infection.

## RESULTS

### Clinical characteristics of *pks*-positive and *pks*-negative *K.pneumoniae* isolates.

**FIG 1:**
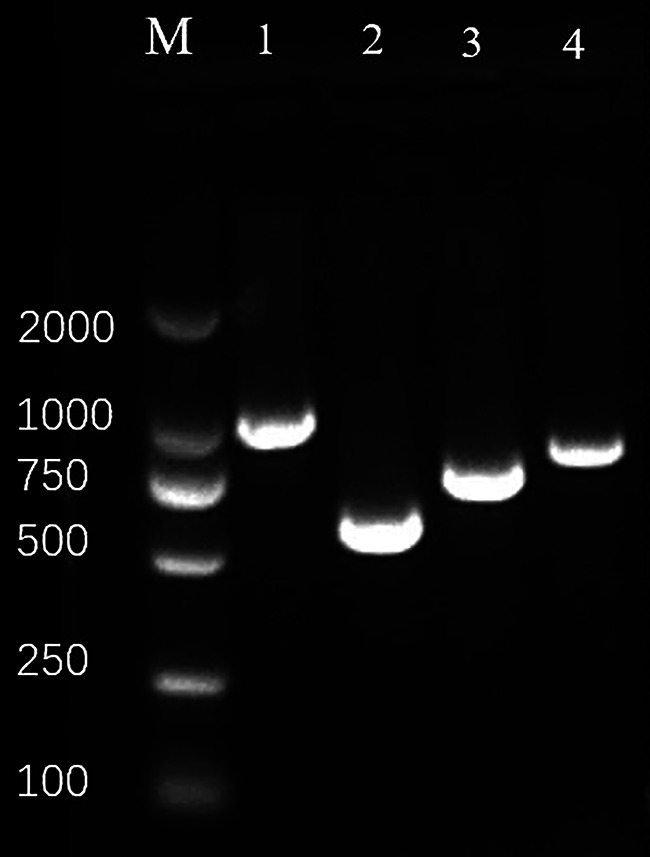
Results of agarose gel electrophoresis of *clbA*, *clbB*, *clbN*, and *clbQ*. Lanes: M, DNA molecular weight standard; 1, *clbA*, 1,311 bp; 2, *clbB*, 579 bp; 3, *clbN*, 733 bp; 4, *clbQ*, 821 bp.

A total of 95 strains of K. pneumoniae were collected and divided into two groups based on the *pks* gene identification results: the *pks*-positive group and the *pks*-negative group. The determination of *pks* gene-positive results was based on the positivity of *clbA*, *clbB*, *clbN*, and *clbQ* (Fig. 1). *pks*-positive K. pneumoniae strains were more likely to infect patients from the emergency department than *pks*-negative K. pneumoniae strains. *pks*-positive K. pneumoniae strains were more often isolated from blood samples (*P* < 0.05). Besides, we found that patients with *pks*-positive K. pneumoniae infection had fewer concomitant underlying diseases than patients with *pks*-negative K. pneumoniae infection (Table 1).

**TABLE 1:** Demographic and clinical data of patients according to the isolation of *pks*-positive and *pks*-negative K. pneumoniae

Characteristic	Result for isolates		*P* value^*a*^
	*pks* positive (*n* = 38)	*pks* negative (*n* = 57)	
Male, no. (%)	29 (76.3)	41 (71.9)	0.634
Age, yr (mean ± SD)	58 ± 2.7217	63 ± 2.4765	0.174
Specimen source, no. (%)			
Sputum	13 (34.2)	40 (70.2)	0.001*
Blood	9 (23.7)	2 (3.5)	0.007*
Pus	7 (18.4)	4 (7.0)	0.169
Bile/abdominal fluid	7 (18.4)	4 (7.0)	0.169
Other	2 (5.3)	7 (12.3)	0.431
Hospital section source, no. (%)			
ICU	6 (15.8)	21 (36.8)	0.026*
Neurology	3 (7.9)	10 (17.5)	0.180
Gastroenterology	11 (28.9)	7 (12.3)	0.075
Emergency	9 (23.7)	3 (5.3)	0.020*
Other	9 (23.7)	16 (28.1)	0.634

a A *P* value of <0.05 was considered to be statistically significant (*).

### Prevalence of *pks* gene cluster, capsular serotypes, and virulence genes.

In this study, all five virulence genes (*peg-344*, *rmpA*, *rmpA2*, *iucA*, and *iroB*) were mainly present in *pks*-positive K. pneumoniae isolates, with higher detection rates than the *pks*-negative K. pneumoniae isolates (*P* < 0.001). The detection rate of serotypes among *pks*-positive K. pneumoniae isolates was 81.6%; the predominant serotypes were K1 (47.4% [18/38]) and K2 (18.4% [7/38]). Six isolates fell into other K serotypes (K5, K20, and K57, with 1, 2, and 3 isolates, respectively), and seven were serologically untypeable (Table 2).

**TABLE 2:** Virulence genes and capsular serotypes of *pks*-positive K. pneumoniae

Virulence factors	No. (%) of isolates		*P* value^*a*^
	*pks* positive (*n* = 38)	*pks* negative (*n* = 57)	
Virulence genes			
*peg-344*	34 (89.4)	21 (36.8)	<0.001*
*rmpA*	34 (89.4)	21 (36.8)	<0.001*
*rmpA2*	32 (84.2)	20 (35.0)	<0.001*
*iucA*	35 (92.1)	23 (40.3)	<0.001*
*iroB*	34 (89.4)	21 (36.8)	<0.001*
Capsular serotypes			
K1	18 (47.4)	1 (1.5)	<0.001*
K2	7 (18.4)	4 (7.0)	0.169
K5	1 (2.6)	4 (7.0)	0.639
K20	2 (5.3)	1 (1.7)	0.719
K54	0 (0.0)	1 (1.7)	1.000
K57	3 (7.9)	4 (7.0)	1.000
NA^*b*^	7 (18.4)	42 (73.6)	<0.001*

a A *P* value of <0.05 was considered to be statistically significant (*).

b NA, not applicable.

### Antimicrobial susceptibility.

Compared with *pks*-negative K. pneumoniae, *pks*-positive K. pneumoniae strains were significantly more susceptible to 10 antimicrobial agents, including ceftazidime, cefepime, aztreonam, imipenem, meropenem, amikacin, tobramycin, levofloxacin, and trimethoprim-sulfamethoxazole (Table 3).

**TABLE 3:** Susceptibility of *pks*-positive and *pks*-negative K. pneumoniae isolates to antimicrobials

Antibiotic	No. (%) of isolates		*P* value
	*pks* positive (*n* = 38)	*pks* negative (*n* = 57)	
CAZ	5 (13.1)	38 (66.6)	<0.001
FEP	5 (13.1)	36 (63.1)	<0.001
ATM	5 (13.1)	38 (66.6)	<0.001
IPM	4 (10.5)	36 (63.1)	<0.001
MEM	4 (10.5)	38 (66.6)	<0.001
AK	4 (10.5)	35 (61.4)	<0.001
TOB	4 (10.5)	32 (56.1)	<0.001
CIP	5 (13.1)	35 (61.4)	<0.001
LVX	5 (13.1)	36 (63.1)	<0.001
SXT	5 (13.1)	28 (49.1)	<0.001

a CAZ, ceftazidime; FEP, cefepime; ATM, aztreonam; IPM, imipenem; MEM, meropenem; AK, amikacin; TOB, tobramycin; CIP, ciprofloxacin; LVX, levofloxacin; SXT, trimethoprim-sulfamethoxazole.

### Biofilm formation.

Our data revealed that 98% of K. pneumoniae isolates were biofilm producers. In this study, 38% and 60% of isolates were weakly and moderately biofilm-producing strains, respectively. The prevalence of moderate biofilm formation in *pks*-positive K. pneumoniae was significantly higher than in *pks*-negative K. pneumoniae (84.2% compared to 43.8%; *P* < 0.05) (Table 4).

**TABLE 4:** Biofilm formation of *pks*-positive and *pks*-negative K. pneumoniae isolates

Clinical isolate type	No. (%) of isolates with biofilm formation model		
	None (0)	Weak (+)	Moderate (++)
*pks* positive	0 (0.0)	6 (15.8)	32 (84.2)
*pks* negative	1 (1.8)	31 (54.4)	25 (43.8)

## DISCUSSION

Klebsiella pneumoniae is a potential hospital superbug that has attracted clinical attention (13). hvKP frequently exhibits hypermucoviscous phenotypes and carries a variety of hypervirulence genes (14, 15). Worryingly, hvKP has been spreading worldwide and causing severe metastatic infections, particularly in immunologically active populations (16). Furthermore, the emergence of multidrug-resistant (MDR) highly pathogenic strains has created significant challenges in the clinical field (17, 18). Therefore, the research on K. pneumoniae could help the clinical treatment of K. pneumoniae-related infection and avoid unnecessary treatment and improper use of medicine.

Two previous surveys pointed out that the proportion of *pks* genes in 207 strains of K. pneumoniae was 25.6% (19) and the proportion of *pks*-positive K. pneumoniae isolates in bloodstream infections was 16.7% (20) in Taiwan. A survey of *pks*-positive K. pneumoniae bloodstream infections in Changsha indicated a prevalence of 26.8% in China (21). Furthermore, the analysis of clinical characteristics showed that *pks*-positive isolates were more frequently encountered in community-acquired infection (21). In this study, the analysis of clinical characteristics showed that *pks*-positive isolates more frequently infected emergency department patients. Compared to *pks*-negative strains, more *pks*-positive strains were collected from blood specimens. These results suggested that the infection of *pks*-positive strains might reflect severe clinical infection.

In this study, we found that the virulence genes were mainly present in *pks*-positive K. pneumoniae isolates, and the positivity rate of the virulence genes (*peg-344*, *rmpA*, *rmpA2*, *iucA*, and *iroB*) was higher than that in the *pks*-negative K. pneumoniae isolates. *peg-344*, *rmpA*, *rmpA2*, *iucA*, and *iroB* were considered to be the biomarkers of highly virulent strains (4). The *peg-344* gene encodes an endometrial transporter and is one of the markers of K. pneumoniae virulence screening (22). *rmpA* and *rmpA2* are regulatory genes for polysaccharide expression in the capsule of K. pneumoniae, which reduce the yield and virulence of the capsule of the strain if missing (23). Iron absorption enhances bacterial virulence. *iucA* and *iroB* are important genes for the expression of K. pneumoniae siderophores, which are major virulence determinants of systemic infection (24). The above findings supported that the *pks* gene cluster might be associated with highly virulent strains.

In this study, the detection rate of highly virulent capsular serotypes in the *pks*-positive K. pneumoniae was 69.5%, with 14 strains not detected. This showed that the positive serotype of the K1 type was significantly higher in *pks*-positive K. pneumoniae strains than in *pks*-negative ones (*P* < 0.05). The presence of capsular serotypes is one of the main virulence factors of hvKP, which can protect the organism against phagocytosis by host phagocytes and damage by lysosomes via their complement (25). Currently, K1 and K2 serotypes of K. pneumoniae can acquire drug-resistant phenotypes by mediating various drug-resistant genes through mobile genetic elements, posing a great challenge for clinical treatment (26). This suggested that the strains carrying a *pks* gene cluster might be more closely associated with virulent capsular serotype K1, and this group of strains might be highly virulent or more likely to acquire a drug-resistant phenotype.

The *pks*-positive isolates were found to be associated with low antimicrobial drug resistance. In this study, statistical analysis showed that *pks*-positive isolates were significantly less resistant to the 10 tested antimicrobial drugs than the *pks*-negative group. This situation might be due to the fact that *pks*-positive isolates have a high proportion of highly pathogenic serotypes and virulence genes, as the acquisition of virulence is usually accompanied by a decrease in resistance. However, we also observed highly resistant strains within the *pks*-positive group, which presents a concerning scenario for the future as it combines genotoxicity and drug resistance. Additionally, our analysis of the data revealed that most *pks*-positive K. pneumoniae isolates exhibited a high capacity for biofilm formation. This biofilm formation might protect bacteria from host immune attack and antibiotics.

Therefore, it is possible that *pks*-positive K. pneumoniae isolates have stronger virulence and pathogenicity, which could result in worse treatment outcomes and prognosis for individuals infected with these strains. To prevent K. pneumoniae infections, there is a need for epidemiological surveillance that targets virulence factors, as well as effective infection control measures and the development of new therapeutic approaches.

## MATERIALS AND METHODS

### Bacterial isolates.

A total of 95 nonrepetitive K. pneumoniae isolates were collected for this study. Relevant clinical data were also retrieved. These isolates were identified, handled, and preserved using standard microbiological laboratory procedures (27).

### Detection of *pks* gene cluster, virulence genes, and capsular serotypes.

The presence of the *pks* gene cluster and virulence genes was detected by PCR as previously described. The clinical isolates were screened for the presence of *pks* gene cluster using primers for the four representative genes (*clbA*, *clbB*, *clbN*, and *clbQ*) of the genomic cluster in order to document the presence of a complete cluster (28). After overnight culture, K. pneumoniae was suspended in 300 μL of sterile distilled water, heated at 95°C for 10 min, and then centrifuged at 12,000 × *g* for 5 min to remove cellular debris. The supernatant was stored at 4°C and used as the template for amplification. The PCR amplification procedure included predenaturation at 94°C for 5 min, denaturation at 95°C for 30 s, annealing at 53°C for 30 s, and 72°C extension for 1 min for 30 cycles, and finally 72°C extension for 10 min. The PCR products were visualized by 2% agarose gel electrophoresis.

To investigate the association of *pks* and hypervirulence, the presence of five hypervirulence genes (*peg-344*, *rmpA*, *rmpA2*, *iucA*, and *iroB*) and capsular serotypes was determined by PCR following previously published protocols (4, 29). The primers used in this study are listed in Table 5.

**TABLE 5:** Primers used in this study

Primer name	DNA sequence (5′→3′)^*a*^	Amplicon size (bp)
*clbA*	CTAGATTATCCGTGGCGATTC	1,311
	CAGATACACAGATACCATTCA	
*clbB*	GATTTGGATACTGGCGATAACCG	579
	CCATTTCCCGTTTGAGCACAC	
*clbN*	GTTTTGCTCGCCAGATAGTCATTC	733
	CAGTTCGGGTATGTGTGGAAGG	
*clbQ*	CTTGTATAGTTACACAACTATTTC	821
	TTATCCTGTTAGCTTTCGTTC	
Virulence genes		
*peg-344*	CTTGAAACTATCCCTCCAGTC	508
	CCAGCGAAAGAATAACCCC	
*rmpA*	TTAACTGGACTACCTCTGTTTCAT	535
	AATCCTGCTGTCAACCAATACT	
*rmpA2*	ATCCTCAAGGGTGTGATTATGAC	447
	CCTGGAGAGTAAGCATTGTAGAAT	
*iucA*	CTCTTCCCGCTCGCTATACT	116
	GCATTCCACGCT TCACTTCT	
*iroB*	GTGAAGTCGATGCCGAGATTATC	199
	CCGAAGACGATCTGTGGAATAC	
Capsular serotypes		
K1	GTAGGTATTGCAAGCCATGC	1,046
	GCCCAGGTTAATGAATCCGT	
K2	GGAGCCATTTGAATTCGGTG	1,121
	TCCCTAGCACTGGCTTAAGT	
K5	GCCACCTCTAAGCATATAGC	999
	CGCACCAGTAATTCCAACAG	
K20	CCGATTCGGTCAACTAGCTT	1,116
	GCACCTCTATGAACTTTCAG	
K54	CATTAGCTCAGTGGTTGGCT	881
	GCTTGACAAACACCATAGCAG	
K57	CGACAAATCTCTCCTGACGA	1,182
	CGCGACAAACATAACACTCG	

a For each primer, the top sequence represents the forward primer and the bottom sequence represents the reverse primer.

### Antibiotic susceptibility.

Antimicrobial susceptibility testing was carried out with bioMérieux Vitek-2 (bioMérieux). The MICs of antimicrobial agents were interpreted according to the guideline established by the Clinical and Laboratory Standards Institute (CLSI) (30). A panel of 10 antimicrobial agents was tested, including amikacin, aztreonam, ceftazidime, ciprofloxacin, cefepime, imipenem, levofloxacin, meropenem, tobramycin, and trimethoprim-sulfamethoxazole.

### Detection of biofilm formation.

Biofilm-forming ability was detected by crystal violet staining. The strains were incubated in LB broth medium, shaken overnight at 37°C, prepared in 0.5 MacConkey's turbidity solution, and diluted 1:100 with LB broth. The diluted broth was added to a 96-well microtiter plate at 200 μL/well, and 3 wells were inoculated with 200 μL sterile LB broth as a negative control. The plate was washed three times with phosphate-buffered saline (PBS [pH 7.0]), dried at room temperature, fixed with methanol solution for 20 min, the methanol was discarded, and then the plate was stained using 1% crystal violet solution. After 15 min, the plate was washed with PBS until colorless. After drying, 200 μL of anhydrous ethanol was used to fully dissolve the crystal violet, the mixture was transferred to a new microplate, and absorbance was measured at 570 nm. Each assay was performed in triplicate and repeated four times.

The optical density cutoff (ODc) was defined as 3 standard deviations (SDs) above the mean optical density (OD) of the negative control. All of the strains were classified based on the adherence capabilities into the following categories: non-biofilm producers (OD ≤ ODc), weak biofilm producers (ODc < OD ≤ 2 × ODc), moderate biofilm producers (2 × ODc < OD ≤ 4 × ODc), and strong biofilm producers (4 × ODc < OD) (31).

### Statistical analysis.

Categorical variables were analyzed by using the chi-square test or Fisher's exact test. For continuous variables, Student's *t* test or the Mann-Whitney U test was used to analyze the data, as appropriate. All data analysis was performed with SPSS software (version 25.0). A *P* value of <0.05 was considered statistically significant.
